# Dysfunction of ventrolateral prefrontal cortex underlying social anxiety disorder: A multi-channel NIRS study

**DOI:** 10.1016/j.nicl.2015.05.011

**Published:** 2015-05-28

**Authors:** Chika Yokoyama, Hisanobu Kaiya, Hiroaki Kumano, Masaru Kinou, Tadashi Umekage, Shin Yasuda, Kunio Takei, Masami Nishikawa, Tsukasa Sasaki, Yukika Nishimura, Naomi Hara, Ken Inoue, Yui Kaneko, Shin-ichi Suzuki, Hisashi Tanii, Motohiro Okada, Yuji Okazaki

**Affiliations:** a Akasaka Clinic for Psychosomatic Medicine and Psychiatry, Medical Corporation Warakukai, Tokyo, Japan; b Department of Psychiatry, Division of Neuroscience, Graduate School of Medicine, Mie University, Mie, Japan; c Faculty of Human Sciences, Waseda University, Saitama, Japan; d Institute of Applied Brain Sciences, Waseda University, Saitama, Japan; e Ohara Clinic for Psychosomatic Medicine, Saitama, Japan; f Division for Environment, Health and Safety, University of Tokyo, Tokyo, Japan; g Neural Plasticity Project, Tokyo Metropolitan Institute of Medical Science, Tokyo, Japan; h Kanagawa Psychiatric Center, Yokohama, Japan; i Office for Mental Health Support, University of Tokyo, Tokyo, Japan; j Department of Social Education, Kawamura Gakuen Women's University, Chiba, Japan; k Laboratory of Health Education, Graduate School of Education, University of Tokyo, Tokyo, Japan; l Department of Neuropsychiatry, University of Tokyo, Tokyo, Japan; m Department of Child Neuropsychiatry, Graduate School of Medicine, University of Tokyo, Tokyo, Japan; n Department of Public Health, Faculty of Medicine, Shimane University, Japan; o Graduate School of Human Sciences, Waseda University, Saitama, Japan; p Tokyo Metropolitan Matsuzawa Hospital, Tokyo, Japan

**Keywords:** Social anxiety disorder, Ventrolateral prefrontal cortex, Emotion, Near-infrared spectroscopy (NIRS)

## Abstract

Social anxiety disorder (SAD) is characterized by strong fear and anxiety during social interactions. Although ventrolateral prefrontal cortex (VLPFC) activity in response to emotional stimuli is related to pathological anxiety, little is known about the relationship between VLPFC activity and social anxiety. This study aimed to investigate whether VLPFC activity was involved in SAD and whether VLPFC activity was related to the level of social anxiety. Twenty-four drug-naïve patients with SAD and 35 healthy controls underwent near-infrared spectroscopy (NIRS) scanning while performing a verbal fluency task (VFT). Results indicated that, compared to the healthy controls, the SAD patients exhibited smaller changes of oxygenated hemoglobin (oxy-Hb) concentrations in the VLPFC during the VFT. Furthermore, the right VLPFC activation was negatively correlated with social avoidance. In contrast to the latter, the healthy controls exhibited a positive correlation between changes of oxy-Hb concentrations in the bilateral VLPFC and social fear. Our findings provide evidence for VLPFC dysfunction in SAD, and indicate that the VLPFC dysfunction may contribute to the difference between normal and abnormal social anxiety.

## Highlights

• We investigated ventrolateral prefrontal cortex (VLPFC) activity induced by the performance of the verbal fluency task.
• Smaller changes of oxygenated hemoglobin (oxy-Hb) concentrations in bilateral VLPFC in SAD patients
• Negative correlation between changes of oxy-Hb concentrations in right VLPFC and social avoidance in SAD patients
• Positive correlation between changes of oxy-Hb concentrations in bilateral VLPFC and social fear in healthy subjects

## 1 Introduction

Social anxiety disorder (SAD) is the most common anxiety disorder (Stein and Stein, 2008). It is characterized by excessive fear and by avoidance of social situations. A core component of social anxiety is the fear of negative evaluation (Clark and Wells, 1995; Rapee and Heiberg, 1997). The lifetime prevalence of SAD has been estimated to be 12% (Kessler et al., 2005a). SAD is often co-morbid with major depressive disorder and bipolar disorder (Kessler et al., 2005b).

Most of the functional brain imaging studies about SAD have showed that emotional reactivity is associated with amygdala hyperactivity in response to social stimuli (e.g. facial expressions, public speaking) (Tillfors et al., 2001; Stein et al., 2002; Ekin and Wager, 2007; Freitas-Ferrari et al., 2010). Amygdala hyperreactivity in SAD patients has also been observed in response to emotional stimuli independently of social cues (Brühl et al., 2011). Moreover, amygdala reactivity in SAD patients has been positively correlated with the severity of social anxiety symptoms (Phan et al., 2006; Evans et al., 2008). Taken together, these results imply that SAD patients have a high level of emotional arousal.

A previous study (Sladky et al., 2012) has reported that when exposed to emotional faces, SAD patients show increased activations of the ventromedial prefrontal cortex (VMPFC) and amygdala. Moreover, during habituation, SAD patients show decreased activations of both the VMPFC and the amygdala. Furthermore, VMPFC activation is known to be involved in the regulation of the amygdala in extinction learning. However, studies of general emotional regulation in healthy participants reported that greater activation in the ventrolateral prefrontal cortex (VLPFC) occurs during reappraisal of negative emotions (Wager et al., 2008; Campbell-Sills et al., 2011). Additionally, decreased activation in the amygdala has been associated with the success of emotional regulation (Wager et al., 2008). A recent study has shown decreased activation in areas adjacent to the VLPFC and VMPFC in response to negative emotional pictures without social cues in SAD patients, compared to healthy participants (Brühl et al., 2011). In the study of generalized anxiety disorder (GAD), more severe GAD symptoms are associated with decreased activation of the VLPFC in response to emotional stimuli (Monk et al., 2006). Although these studies have shown that attenuated VLPFC function is associated with pathological anxiety, the function of the VLPFC in SAD remains unclear. To date, there are no data that would support such an expectation for SAD patients. Furthermore, no prior imaging study of healthy participants has examined the relationships between the VLPFC activation and degree of social anxiety.

In emotional tasks and settings, the method for manipulating emotional response and emotional control has been different in each experiment (Ochsner and Gross, 2005; Wager et al., 2008; Van Dillen et al., 2009; Clarke and Johnstone, 2013). The manipulations involve either using an emotional induction or using instructed emotional control. The pattern of activation (i.e., increase or decrease) in response to emotion is very likely to differ for these different manipulations (Ochsner and Gross, 2014). Therefore, the current study used neither an emotional task inducing strong emotional states nor instructed emotional control. By assessing the brain activity in response to a verbal fluency task (VFT), which will evoke the fear of evaluation by others (Bögels et al., 2010), this study will evaluate the brain activity related to emotional response and emotional control (Dolcos et al., 2011; Pessoa, 2013).

The present research was designed to compare cerebral hemodynamic responses in the VLPFC during the VFT between SAD patients and healthy controls by employing multi-channel near-infrared spectroscopy (NIRS). In addition, within each group, the relationship between the hemodynamic responses in the VLPFC during the VFT and the degree of social anxiety was also examined. Our hypothesis was that the VLPFC is activated to a smaller extent in the SAD patients than in the healthy controls. Also, we predicted that in the SAD patients, the VLPFC activation is negatively correlated with the degrees of social anxiety. In the healthy controls, there may be no correlation or a positive correlation between the VLPFC activation and the degree of social anxiety.

## 2 Methods

### 2.1 Participants

Twenty-four drug-naïve patients with SAD and 35 age- and gender-matched healthy controls participated in this study. The demographic and clinical data for participants are shown in Table 1. The patients were recruited from Warakukai Medical Corporation's clinics. These clinics specialize in the treatment of anxiety and depressive disorders in Japan. All patients were diagnosed with SAD according to DSM-IV-TR criteria by psychiatrists (HK, TU, SY, KT, MN, and TS). Six patients met the criteria for non-generalized subtype of SAD. One patient had a co-morbid agoraphobia, but the others did not have any other anxiety disorders. The Mini-International Neuropsychiatric Interview confirmed that none of the healthy controls met the diagnostic criteria for SAD, or other psychiatric disorders. The healthy controls had no history of psychiatric disorders in their first-degree relatives. Inclusion criteria for the participants were physical health and right handedness, as assessed using the Edinburgh Handedness Inventory (score > +70; Oldfield, 1971). Exclusion criteria for the participants were a history of neurological disorder, a previous head trauma, a current or past history of inpatient psychiatric care, any intake of medication for anxiety or depression, and any history of substance abuse. In addition, patients with current depression or mania were excluded. All participants completed Spielberger's State–Trait Anxiety Inventory (STAI) (Spielberger et al., 1983), the Profile of Mood States (POMS) short form (Lorr et al., 1971), the Zung Self-rating Depression Scale (SDS) (Zung, 1965) and the Liebowitz Social Anxiety Scale (LSAS) (Liebowitz, 1987). The LSAS has been developed for assessing fear and avoidance associated with social anxiety. The state component of the STAI was administered before and after the NIRS scanning. The POMS assesses the mood states of tension, depression, anger, vigor, fatigue and confusion. The scale was used with instructions requesting participants to state how they feel “right now,” and was administered immediately after NIRS scanning.

The Ethics Committee of Mie University and Warakukai Medical Corporation approved this study. Written informed consent was obtained from all participants before they enrolled in the study. Participants were also informed that they were free to withdraw from the study at any time without any negative consequences.

### 2.2 Verbal fluency task

The verbal fluency task (letter version) consisted of a 30 s pre-task (baseline), a 60 s activation (VFT) task, and a 60 s post-task. During the activation task period, the participants were instructed to generate as many Japanese words beginning with a designated syllable as possible. The three sets of initial syllables (first; /to/, /se/, /o/, second; /a/, /ki/, /ha/, third; /na/, /i/, /ta/) were presented in counterbalanced order among the participants; each presented syllable changed every 20 s during the 60 s activation task. Because the number of words generated by the three sets was not significantly different (mean ± SD: first, 5.05 ± 1.74; second, 4.97 ± 1.78; third, 4.34 ± 1.96 words; *F*[2,56] = 2.67, n.s., in a one-way ANOVA using sets as an independent variable), the total number of correct words was defined as the measure of task performance. During the pre-task and post-task periods, the participants were instructed to repeat a train of Japanese vowel syllables (/a/, /i/, /u/, /e/, /o/) aloud. This was intended to match possible vocalizing effects in the baseline and activation task periods.

### 2.3 NIRS measurement

**Fig. 1:**
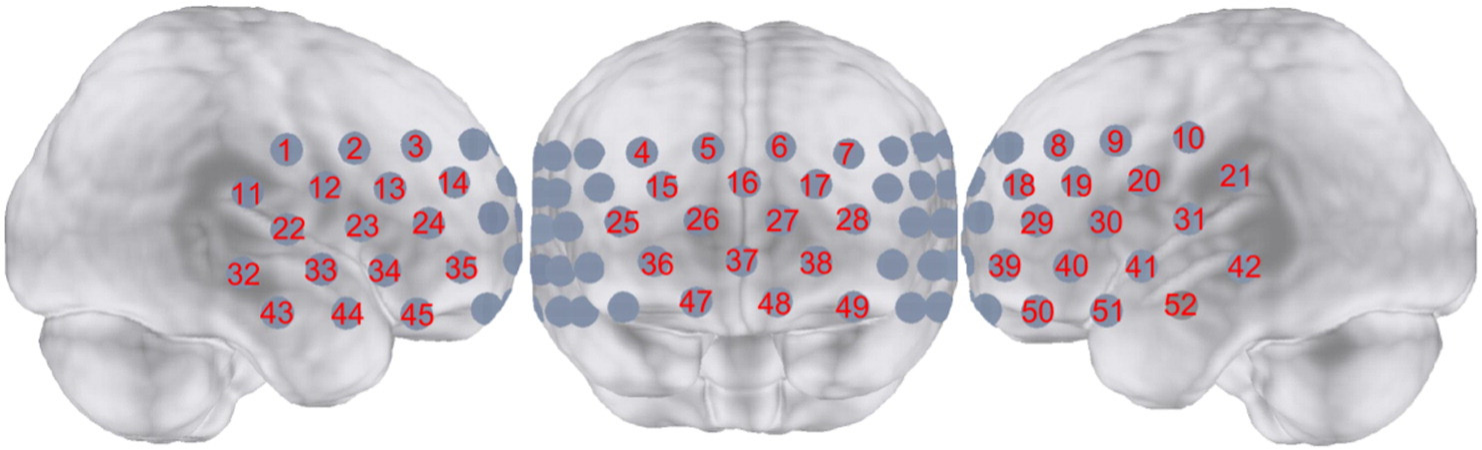
Probe setting and measurement points for 52-channel near-infrared spectroscopy (NIRS). The channel numbers are indicated above the cortical regions.

We used an ETG-4000 (Hitachi, Japan) Optical Topography system to measure changes in cerebral oxygenated hemoglobin (oxy-Hb) and deoxyhemoglobin (deoxy-Hb) concentrations at 2 wavelengths of near-infrared light (695 and 830 nm). The measurement principles were based on the modified Beer–Lambert law, which calculates changes in oxy-Hb and deoxy-Hb concentrations from the light attenuation change at a given measurement point. By assuming the differential pathlength factor, the changes in oxy-Hb are expressed as oxy-Hb concentration multiplied by average pathlength whose unit is mmolar・mm (Ferrari and Quaresima, 2012). We used a single “3 × 11” measurement patch provided by Hitachi. In the patch, 17 emitters and 16 detectors are alternatingly positioned, for a total of 33 probes. The interprobe distance was 30 mm. Each measurement area between pairs of emitter–detector probes was defined as a ‘channel’ (CH), resulting in a 52-channel measurement. The patch was placed symmetrically on each participant's forehead. Specifically, the lowest probes were placed at positions Fp1 and Fp2 of the international 10–20 electrode system. The arrangement of the probes could measure the changes of oxy-Hb concentration from the bilateral prefrontal (approximately dorsolateral and medial [Brodmann's area (BA) 9, 46], ventrolateral [BA44, 45, 47], and frontopolar [BA10]) and temporal cortical surface regions (Fig. 1).

Practice trials of the VFT were performed prior to the NIRS scanning. Instructions for the experiments were presented via a video on a computer display in front of the participants. The participants were required to take a seat in a space surrounded by partitions, and to avoid making any bodily movements. They were also asked to keep their eyes on a gaze point to minimize the effects of eye movement during the NIRS scanning.

### 2.4 NIRS data analysis

NIRS data were corrected as proposed in previous studies (Nishimura et al., 2007; Takizawa et al., 2008). We chose to only use the oxy-Hb data because the correlations with cerebral blood flow have been shown to be stronger for oxy-Hb than for deoxy-Hb (Malonek et al., 1997; Strangman et al., 2002). The rate of data sampling was 0.1 s. We applied automatic rejection methods for data with artifacts. The rejection methods were applied separately for each channel (Takizawa et al., 2008). The baseline data were calculated using the integral mode; the pre-task baseline was determined as the mean over a 10 s period just prior to the task period, and the post-task baseline was determined as the mean over the last 5 s of the post-task period; linear fitting was applied to the data between these two baselines. A moving average method using a window width of 5 s was applied to remove any short-term motion artifacts. The data of changes in oxy-Hb concentration for use in statistical analysis were obtained by subtracting the baseline data calculated using the integral mode from the original measurement data in oxy-Hb. To estimate the cortical localization of each channel, we used the virtual registration method (Singh et al., 2005; Tsuzuki et al., 2007; Okamoto et al., 2009), which enables the probabilistic registration of NIRS data onto the Montreal Neurological Institute (MNI) coordinate space. According to our working hypotheses, the regions of interest (ROIs) were placed over the bilateral VLPFC as follows: right VLPFC ROI: CH34 and CH45; and left VLPFC ROI: CH40 and CH50. An additional ROI corresponding to the region of the dorsomedial prefrontal cortex (DMPFC) was chosen to estimate emotional reactivity related to the VFT. DMPFC is strongly activated when one's own emotional states are assessed (Fossati et al., 2003). We placed the DMPFC ROI on the CH5 and CH6 channels.

Changes of oxy-Hb concentration in the DMPFC ROI were calculated by averaging changes of oxy-Hb concentration in the CH5 and CH6 channels for the pre-task (a 10 s period just before the VFT was started) and task (60 s) periods for each subject. The resulting data were analyzed by a group (SAD vs. HC) × task-period (pre-task vs. task) two-way repeated-measures analysis of variance (ANOVA). Post hoc comparisons were conducted using a one-way ANOVA. Spearman's rank correlation coefficients were calculated between the changes of oxy-Hb concentration for the task period in the DMPFC ROI and the POMS scores (six subscales), STAI-state scores and LSAS scores within each group.

Group comparisons of each of the 52 channels were performed. For each subject, the change of oxy-Hb concentration in each channel was calculated for the task period. The resultant data were compared between the groups using Student's *t*-test (SAD vs. HC) in all 52 channels. Statistical significance was set at *p* < .05, adjusted by a false discovery rate (FDR) (Singh and Dan, 2006) correction for multiple comparisons.

We also performed an analysis of ROIs. For each subject, the change of oxy-Hb concentration in each VLPFC ROI was calculated by averaging the changes of oxy-Hb concentration of the configured channels in each ROI for the task period. The resultant data were compared between the groups using Student's *t*-test (SAD vs. HC) in the right and left VLPFC ROIs. The threshold of significance was uncorrected *p* = .05. Furthermore, Spearman's rank correlation coefficients were calculated between the changes of oxy-Hb concentration for the task period in the VLPFC ROIs and the LSAS scores (two subscales), STAI scores (two subscales), POMS scores (six subscales), and task performance within each group. The tests for group comparison of the correlation coefficients were conducted by the Fisher z-transformation. The statistical analyses were performed using SPSS 15.0J for Windows.

## 3 Results

### 3.1 Behavioral data

The number of generated words during the VFT for the SAD group (mean ± S.D. = 14 ± 5.0 words) was not significantly different from that for the HC group (mean ± S.D. = 14.52 ± 3.50 words; Student's *t* = –.46, n.s.). That is, the task performance was equivalent for the two groups. Additionally, no significant association was found between task performance and LSAS scores in any group.

### 3.2 NIRS data results

#### 3.2.1 Emotional state data

The two-way repeated-measures ANOVA analyzing changes of oxy-Hb concentration in the DMPFC ROI revealed a significant interaction of “group” and “task-period” (*F* = 4.320, *df* = 1,54, *p* = .042). A simple effect test revealed that changes of oxy-Hb concentration in the DMPFC ROI for the task-period were significantly greater than that for the pre-task period in both the groups (SAD; *F* = 15.139, *df* = 1,42, *p* = .0004, HC; *F* = 4.664, *df* = 1,66, *p* = .034). The changes of oxy-Hb concentration in the DMPFC ROI for the task-period were significantly greater in the SAD group than in the HC group (*F* = 4.127, *df* = 1,54, *p* = .047), whereas no group differences were found for the pre-task period. Positive correlations between changes of oxy-Hb concentration in the DMPFC ROI during the VFT and confusion subscale scores of POMS were found in both the SAD and HC groups (SAD group; *r* = .598, *p* = .003, HC group; *r* = .430, *p* = .011). No significant associations were found between changes of oxy-Hb concentration in the DMPFC ROI and other subscale scores of the POMS, such as tension, depression, anger, vigor and fatigue, STAI state or trait anxiety scores, or the LSAS-fear and avoidance scores. Therefore, it was probable that negative emotions, such as confusion were evoked during the VFT in both groups.

#### 3.2.2 Channel-wise analysis

**Fig. 2:**
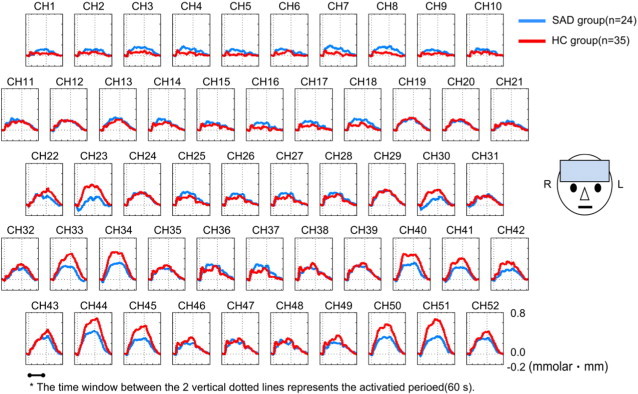
Grand averaged waveforms of changes in oxy-Hb concentration in patients with social anxiety disorder (SAD; blue line) and healthy controls (HC; red line). No significant group differences were observed in all channels (CH).

The grand averaged waveforms of the changes of oxy-Hb concentration during the VFT in the SAD group and the HC group are shown in Fig. 2. The changes of oxy-Hb concentration during the VFT in 6 channels (CH23, 33, 34, 45, 50, 51) were smaller in the SAD group than in the HC group (*t* = −3.127–−2.132, uncorrected *p* = .0032–.0395). These channels excluding CH33 were fully, or partially located in the VLPFC (BA44, 45, and 47). The position of CH33 corresponded to the superior temporal gyrus (BA22). On the other hand, the changes of oxy-Hb concentration during the VFT in 3 channels (CH5, 7, 8) were greater in the SAD group than in the HC group (*t* = 2.232–2.368, uncorrected *p* = .0232–.0308). The three channels were partially located in the dorsomedial PFC (medial BA9) or frontal eye field (BA8). However, the results for these 9 channels were not significant after FDR correction for multiple comparisons.

#### 3.2.3 VLPFC ROI analysis

**Fig. 3:**
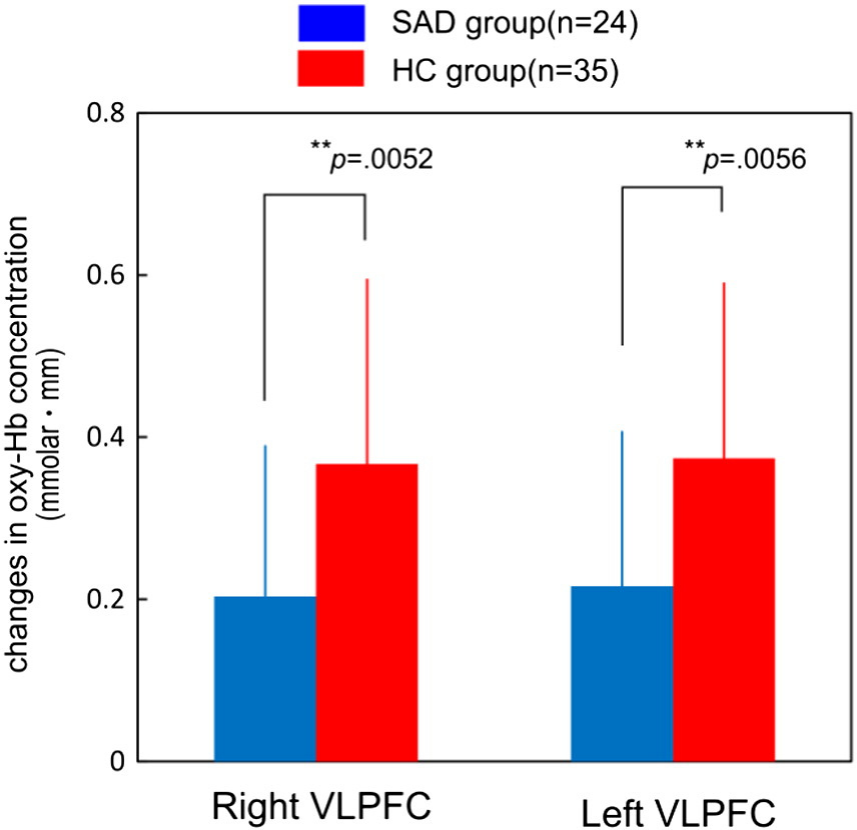
Comparisons of the changes of oxy-Hb concentration in the ventrolateral prefrontal cortex (VLPFC) between patients with social anxiety disorder (SAD) and healthy controls (HC). The changes of oxy-Hb concentration in SAD patients in the VLPFC were significantly smaller than that in controls (**p < .01).

The changes of oxy-Hb concentration during the VFT in the bilateral VLPFC were significantly smaller in the SAD group than in the HC group (right; *t* = –2.901, *p* = .0052, left; *t* = –2.881, *p* = .0056) (Fig. 3).

**Fig. 4:**
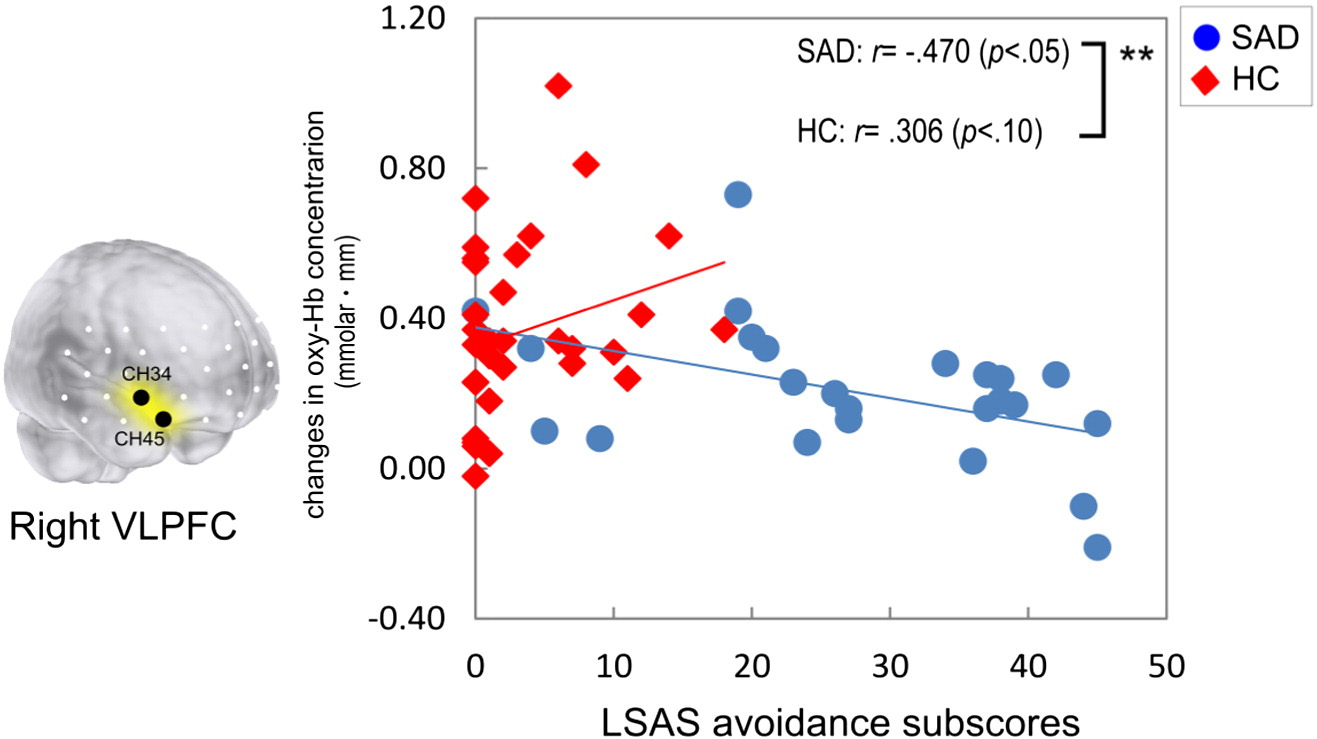
The correlations between the avoidance subscale scores of Liebowitz's Social Anxiety Scale (LSAS) and the changes of oxy-Hb concentration in the right ventrolateral prefrontal cortex (VLPFC) in patients with social anxiety disorder (SAD; blue line and blue circles) and healthy controls (HC; red line and red diamonds). Asterisks in the upper right corners indicate significant group differences of test for the correlation coefficients by the Fisher z-transformation (**p < .01).

For the SAD group, correlation analyses showed that the changes of oxy-Hb concentration in the right VLPFC were negatively correlated with the LSAS-avoidance score (*r* = –.470, *p* = .020). For the HC group, the changes of oxy-Hb concentration in the VLPFC were positively correlated with the LSAS-fear scores (right; *r* = .389, *p* = .025, left; *r* = .419, *p* = .015) and LSAS-avoidance scores (right; *r* = .306, *p* = .083). The group comparisons of the correlation coefficients between the changes of oxy-Hb concentration in the right VLPFC and the LSAS-avoidance scores were significant (z = –2.69, *p* < .01) (Fig. 4). The correlation pattern of the two groups was statistically different in the opposite direction in that the correlation coefficient of the SAD group was negative, whereas that of the HC group was positive.

On the other hand, no significant associations were found between the changes of oxy-Hb concentration in the VLPFC and the STAI state or trait anxiety scores, and the task performance.

## 4 Discussion

The aim of the present study was to compare the changes of oxy-Hb concentration in the VLPFC during the VFT for SAD patients versus those for healthy controls. Consistent with our hypothesis, the SAD patients showed the smaller VLPFC activation during the VFT compared to the healthy controls. Moreover, the SAD patients showed a negative correlation between the right VLPFC activation and the severity of avoidance symptoms, whereas the healthy controls exhibited a positive correlation. The comparison of the correlation of the SAD patients and that of the healthy controls was significant. To the best of our knowledge, this is the first study to demonstrate the differential functional pattern of the PFC in normal and pathological social anxiety.

Previous research (Brühl et al., 2011) has reported reduced activation in the area adjacent to the VLPFC and VMPFC in response to negative emotional pictures in SAD patients, compared to healthy controls. We also found a similar decreased VLPFC activation in SAD patients. Also, a negative correlation between right VLPFC activation and social avoidance was found in SAD patients. These findings are in line with a previous study of GAD patients showing a negative correlation between VLPFC activation and the severity of pathological anxiety (Monk et al., 2006). Additionally, a previous study of adolescents with SAD (Guyer et al., 2008) has showed a positive correlation with connectivity between the VLPFC and the amygdala and the severity of anxiety during anticipated peer evaluation. Taking these former studies along with the present results into consideration suggests that SAD patients with more severe symptoms may show a decreased VLPFC activation to emotional stimuli as opposed to the enhanced connectivity between the VLPFC and the amygdala. In the present study, we found no correlations between VLPFC activity and the degree of STAI state and trait anxiety in SAD patients. Therefore, VLPFC dysfunction might contribute to social anxiety, but it might not reflect general state or trait anxiety. As the VLPFC is involved in the integration of emotional information and the regulation of emotional responses (Cabeza and Nyberg, 2000; Wager et al., 2008), a VLPFC dysfunction might impede emotional processing in SAD patients (Clark and Wells, 1995).

We also found that the healthy participants with higher degrees of social anxiety showed an increased VLPFC activation. One study of healthy participants (Campbell-Sills et al., 2011) reported that anxiety-prone participants compared with healthy controls showed greater activation of the VLPFC during the regulation of negative emotions. Furthermore, greater VLPFC activation was associated with greater reduction of subjective distress in anxiety-prone participants. These findings partially support our findings and suggest that healthy participants with a higher degree of social anxiety might require greater engagements of VLPFC activity to reduce negative emotions.

The present research was conducted without evoking strong negative emotions. For GAD patients, the previous study by Monk et al. (2006) reported a negative correlation between VLPFC activation and the severity of pathological anxiety by evoking strong negative emotions (Monk et al., 2006). As we consider this along with the present results, the relationship between VLPFC activation and the degree of pathological anxiety is constantly negative irrespective of the intensity of evoked negative emotions. Therefore, the abnormal brain functions in these anxiety patients may be characterized by VLPFC dysfunction. Furthermore, the participants in this study were not explicitly instructed to regulate their negative emotions. A previous study (Wager et al., 2008) reported greater right VLPFC activation when healthy participants were instructed to reduce their negative emotions. As we consider this with the present results, the right VLPFC activation may be useful for reducing negative emotions in healthy participants irrespective of the explicit instruction to regulate their emotions. On the other hand, the SAD patients probably fail to regulate their negative emotions because the right VLPFC activity is lower and negatively correlated to the severity of social avoidance. We still need to investigate right VLPFC activity when SAD patients are explicitly instructed to regulate the negative emotions induced by extreme emotional stimuli without social cues.

The present findings suggest that right VLPFC dysfunction may contribute to increasing the clinical severity of SAD. An interesting possibility is that right VLPFC dysfunction can be used as a neurobiological marker of SAD. To date, there are no good objective indicators that could distinguish between normal and abnormal social anxiety and therefore the right VLPFC dysfunction could be useful. Future work is necessary to clarify the relationship between the right VLPFC activity and treatment responses of SAD patients.

The results of this study are constrained by certain limitations. Firstly, patients that participated in this study included only those with mild, or moderate SAD, as assessed by the LSAS total score (average = 62.9, SD = 26.1). Therefore, the VLPFC function in SAD patients with severe symptoms was not explored in this study. Moreover, participants with other anxiety disorders, or mood disorders were excluded from this study. Nevertheless, our findings are consistent with previous studies of GAD, and bipolar disorder patients showing abnormal functioning in the VLPFC (Monk et al., 2006; Foland-Ross et al., 2012). Second, reduced activation of the VLPFC in SAD patients might have been caused by participants with poor executive function. However, there was no difference in task performance between the SAD group and healthy controls. Additionally, the task performance was not significantly associated with the degrees of social anxiety in the SAD group. Thirdly, it is unclear whether there was any amygdala activation. We interpreted our findings based on prior studies of the amygdala and VLPFC function in healthy subjects and GAD patients.

## 5 Conclusions

In conclusion, we provide evidence of the abnormal VLPFC activity in SAD. The findings have important implications for understanding the social brain in humans as well as the functional abnormalities in pathological anxiety. However, it remains an open question whether and how the abnormal VLPFC activity influences other cortical and subcortical brain activities. This challenge must be tackled to improve our understanding of functional abnormalities of SAD.

## Conflict of interest

This work was supported in part by KAKENHI (a Grant-in-Aid for Scientific Research) in the Priority Areas “Applied Genomics” (YO) (No. 17019029) and KIBANKEISEI (the Strategic Research Platforms for Private University : Matching Fund Subsidy) in 2010 (HK) from the Ministry of Education, Culture, Sports, Science, and Technology of Japan.

## Figures and Tables

**Table 1:** Demographic and clinical characteristics in patients with social anxiety disorder (SAD) and healthy controls (HC).

	SAD group (n = 24)	HC group (n = 35)		
	Mean ± SD	Mean ± SD	*t*-Value	*p*-Value
Gender, women/men	12/12	18/17	–	. 91^a^
Age, years	36.3 ± 12.8	37.3 ± 10.9	−0.34	.74
Education, years	14.6 ± 2.0	16.2 ± 1.7	−3.20	<.01
Estimated IQ (JART-25)	104.0 ± 10.5	104 ± 9.7	0.01	.99
LSAS				
Total scores	62.9 ± 26.1	17.5 ± 20.3	7.52	<.001
Fear	35.5 ± 15.1	12.1 ± 11.0	6.49	<.001
Avoidance	27.5 ± 13.5	5.4 ± 10.0	7.22	<.001
STAI				
STAI-T	50.2 ± 11.9	35.5 ± 7.5	5.32	<.001
STAI-S				
Pre-scanning	42.7 ± 8.3	37.1 ± 8.9	2.45	<.05
Post-scanning	40.0 ± 9.3	35.6 ± 8.4	1.86	.07
POMS				
Tension	10.0 ± 4.9	4.0 ± 4.0	4.92	<.001
Depression	6.9 ± 5.0	1.9 ± 2.5	5.00	<.001
Anger	4.4 ± 3.4	2.0 ± 1.8	3.52	<.001
Vigor	6.3 ± 5.5	9.8 ± 4.4	−2.58	<.05
Fatigue	8.0 ± 4.5	4.7 ± 4.1	2.83	<.01
Confusion	6.3 ± 4.2	3.8 ± 2.6	2.86	<.01
SDS	42.1 ± 9.4	32.2 ± 6.3	4.86	<.001

JART-25: Japanese version of the National Adult Reading Test. LSAS: Liebowitz Social Anxiety Scale. STAI: State–Trait Anxiety Inventory. POMS: Profile of Mood States. SDS: Zung Self-rating Depression Scale.

a Chi-square test, otherwise *t*-test was used for testing group difference.
